# Intra-Articular Laser Treatment Plus Platelet Rich Plasma (PRP) Significantly Reduces Pain in Many Patients Who Had Failed Prior PRP Treatment

**DOI:** 10.3390/medicines6030075

**Published:** 2019-07-16

**Authors:** Chadwick C. Prodromos, Susan Finkle, Alexander Dawes, Angelo Dizon

**Affiliations:** 1 Illinois Sportsmedicine & Orthopaedic Centers, 1714 Milwaukee Ave, Glenview, IL 60025, USA; 2 Advanced Physical Therapy Institute, 1714 Milwaukee Ave, Glenview, IL 6002, USA

**Keywords:** platelet rich plasma, low level laser, osteoarthrosis, intra-articular laser

## Abstract

**Background:** In our practice, Platelet Rich Plasma (PRP) injections effectively reduce pain in most, but not all, arthritic patients. When PRP treatment fails, joint replacement surgery is often the only good alternative. Surface Low-Level-Laser-Therapy (LLLT) has not been helpful for osteoarthrosis in our experience. We hypothesized that intra-articular laser (IAL) treatment combined with PRP would improve results in patients with prior ineffective PRP treatment. **Methods:** We offered Intra-articular Low-Level-Laser-Therapy (IAL) treatment simultaneously with repeat PRP injection to patients who had received no benefit from PRP alone. They were the treatment and also historical control group since all had failed PRP treatment alone. Thirty joints were treated: 22 knees, 4 hips, 2 shoulder glenohumeral joints and 2 first carpo-metacarpal (1st CMC). **Results:** No adverse events were seen at any time after treatment in any patient. Twenty-eight joints were available for re-evaluation: ≥ 40% improvement was seen in 46% (6 months), 32% (12 months) and 32% (24 months) post-treatment. Mean SANE scores improved significantly at 1 and 2 years. Thirteen patients failed treatment and had joint replacement. **Conclusions:** PRP with IAL allowed avoidance of surgery and good pain control at least two years post-treatment in nearly half of patients who had failed PRP treatment alone.

## 1. Introduction

In our practice, Platelet Rich Plasma (PRP) injections effectively reduce pain in most but not all arthritic patients. However, for patients who fail PRP treatment, no good alternative currently exists, except joint replacement surgery. We have not found second PRP injections to reduce arthrosis symptoms if a first injection has failed. Low Level Laser Therapy (LLLT) has been shown in animal studies to modulate inflammatory pathways and stimulate tissue metabolism both on its own [1] and to a greater extent in conjunction with PRP [2,3]. Studies of treatment of knee osteoarthrosis with LLLT in humans have had mixed results with us and others [4,5] finding it ineffective, while others have reported success [6,7,8,9,10,11,12]. It has been postulated that the negative results may be due to the fact that laser irradiation can only travel short distances through tissue, reducing the effects inside larger joints [4]. Weber has suggested moving the laser probes from the surface of the skin to the interior of the joint in order to allow the light to reach the desired tissues and create an effective treatment of arthrosis [13]. We therefore hypothesized that intra-articular laser treatment (IAL) might be an effective augmentation to PRP injection and might increase the efficacy of a subsequent PRP injection in patients who had failed prior PRP injection alone.

## 2. Materials and Methods

Between July 2016 to June 2017, we offered IAL in conjunction with repeat PRP injection to patients with arthrosis who had received no benefit from PRP injection alone. All were surgical candidates and sought PRP as an alternative to surgery. They were the treatment group. They were not charged for PRP or IAL. They also served as a historical control group since they had all failed PRP treatment alone. All subjects gave their informed consent for inclusion before they participated in the study. The study was conducted in accordance with the Declaration of Helsinki, and the protocol was approved by the IRB of the Foundation for Orthopaedics and Regenerative Medicine (2016-01). 

Forty-five mL of blood was drawn from each patient and processed sterilely through a double spin technique to create one 4 mL dose of PRP with a platelet count roughly quintuple that of the whole blood. According to the PAW classification system by DeLong [14], the PRP preparation was P3-Aα. The patient’s affected joint was sterilely prepped and two Weber intra-articular needles (Weber Medical: http://www.webermedical.com/en/contact) of 4, 6 or 8 cm length, depending on the joint injected and the girth of the patient, were inserted at two sites within the same joint using sterile technique and local subcutaneous, but not intra-articular, lidocaine infiltration. Needles were placed into the joint space so that they could bathe the entire space with light. Placement of the needles varied depending on the treated area. In knees with patellofemoral disease, the two needles were placed into the patellofemoral space from a medial approach. For knees with medial or lateral symptoms, the needles were placed from a parapatellar approach, one medially and one laterally. In shoulders, both needles were placed in the glenohumeral joint from a posterior approach. For hips, both needles were placed in the acetabulofemoral joint from an anterior approach lateral to the femoral neurovascular structures. For the first carpo-metacarpal joints, one needle was placed into the joint from a posterior approach and one from an anterior approach. Ultrasound guidance was used in the placement of all needles. The PRP was then injected into one of the needles. Two Weber intra-articular laser flexible wires were inserted, one into each hollow needle. Each site had 10 min of red (658 nm), infra-red (810 nm) and blue (405 nm) laser radiation, for a total of 30 min illumination time, at 100 MW. The patients sat or reclined during the procedure. The needles were removed when the procedure was completed. The senior author (CP) performed all procedures.

Before treatment, all patients completed a SANE pain score and a joint-specific survey (KOOS-PS & Womac for knees, Quick DASH for shoulders and 1st CM joints, and NAH score for Hips). Patients were followed up at 1 week, 1 month, 6 months, 1 year, and 2 years after treatment. They were asked to provide a percent better or worse than before treatment and completed a SANE score at each follow-up. At 6 months, 1 year, and 2 years, joint specific surveys were collected as well. 

## 3. Results

Twenty-eight patients (30 joints) accepted treatment and 22 knees (20 patients), 4 hips, 2 shoulder glenohumeral joints, and 2 first carpo-metacarpal (1st CMC) joints were treated (see Table 1 for patient demographics). All patients had radiographic and clinical evidence of osteoarthrosis. Of the 22 knee joints, standing X-rays showed that in the treated compartment, 8 of them were bone on bone, 4 had 1 mm of joint space, 1 had 2 mm, 3 had 3 mm, 2 had 4 mm, and 4 had 5 mm of joint space. For the shoulder joints, Grashey (true anterior-posterior view) views showed that one joint was bone on bone and one had 1 mm of joint space. For the hip joints, 1 was classified as Tonnis grade 2 and 3 were Tonnis grade 3. Thirteen joints had received 1 prior PRP injection without improvement in symptoms. Seventeen joints had received two or more previous injections with only transient relief. Of the 17, all received at least 1 injection of PRP. Five had a second PRP injection, five had a second injection of Synvisc, and the remaining six had between 3 and 8 previous injections of PRP and Synvisc. The mean age of the patients was 67 years (range 54–88) and the mean BMI was 29.0 (range 18.6–40.2). Fourteen were male and 12 were female (14 joints). Treatment was performed on 18 right joints and 12 left joints. 

All patients were seen 1 week post-treatment and there were no adverse events in any patient. 

Two patients were lost to follow up (1 knee and 1 shoulder), one after 1 month, one after 4 months. All other patients (93%) had follow-up out to at least 2 years. At 6 months, 13 of 28 joints (46%) had a good result: 9 of 21 knees, 2 of 4 hips, 0 of 1 shoulder and 2 of 2 1st CMC joints. At 1 year, 9 of 28 joints remained improved from before treatment (32%): 6 knees, 1 hip, 0 shoulders, and 2 1st CMC joints. Joints that were good at 1 year remained good at 2 years, with 32% continuing to show improvement. A good result was defined as a percent improvement of at least 40%, normal function with activities of daily living and avoidance of joint replacement surgery. Thirteen joints had joint arthroplasty performed after the treatment: 9 knees, 3 hips, and 1 shoulder. Mean time to replacement was 10.2 months after treatment.

Mean SANE scores before treatment for all treated joints were 61.1 (scale 0–100, 100 worst). Mean 6 month scores were 46.4, 1 year scores were 41.3 and 2 year scores were 35.5. The differences between pretreatment scores and post treatment scores were significantly different at 1 year (*p* = 0.04) and 2 years (*p* = 0.01), but not at 6 months (*p* = 0.06). (Raw data for all patients can be found in supplement File S1)

### 3.1. Knees

Nine of the 21 knee joints were improved at 6 months (43%), and 6 were improved at 1 year and at 2 years (29%). Patients with knee pathology were injected into the compartment where their pain was localized. Sixteen had medial compartment pain, 2 had lateral compartment pain, and 4 had patellofemoral pain. Of the joints with medial joint pain, 43.8% were improved at 6 months and 25% were improved at 1 year and 2 years. Of the joints with patellofemoral pain, 67% showed improvement at 6 months, 1 year and 2 years. Neither of the joints with lateral pain showed improvement at any follow-up point. (See Table 2 for a summary of the knee results.)

Knee joints were also classified by joint space into two groups based on standing X-rays, those with bone-on-bone (BOB) joints or 1 mm of remaining space and those with 2 mm or more of joint space. Forty percent of the group with 2 mm or more of joint space showed improvement at all follow-up intervals. In the BOB/1 mm joint space group, 46% were improved at 6 months, and 18% were improved at 1 year and 2 years. 

Joint replacement was performed in 9 of the 21 treated knee joints by the 2 year follow up. Eight of these joints had medial pathology, 1 had lateral pathology. No joint replacements occurred in the patellofemoral group. Seven of the joint replacements were in patients in the BOB/1 mm joint space group (64% of this group) and 2 joint replacements were in the 2 mm or greater group (20% of this group). 

Mean SANE scores before treatment for all knees were 68.3. At 6 months, the mean score was 50.1, at 1 year 40.0, and at 2 years 35.0. The pre and post treatment scores were significantly different for all time periods (6 mo *p* = 0.02, 1 year *p* = 0.01, 2 years *p* = 0.003). Mean KOOS-PS scores changed from a mean of 50.6 pre-treatment to 65.9 at 6 months, 65.6 at 1 year, and 66.8 at 2 years (scale 0–100, 0 worst). Mean pre- and post-treatment scores were significantly different at 6 months and 1 year (*p* = 0.03 for both) but not at 2 years (*p* = 0.06). WOMAC scores had a mean of 16 before treatment and 20.8 at 6 months, 22 at 1 year, 24.9 at 2 years (scale 0–96, 96 worst). The pre and post scores were significantly different at all time periods (6 mo *p* = 0.01, 1 year *p* = 0.005, 2 years *p* = 0.01). 

### 3.2. Shoulders

The one treated shoulder for which follow-up was available had 1 mm of joint space as measured on a Grashey view X-ray. This shoulder showed no improvement from the treatment. Joint replacement was performed 23 months after initial treatment. 

### 3.3. Hips

Two of 4 of the hips were improved at 6 months (50%) and 1 was improved at 1 year and 2 years (25%). Treated hip joints were separated into 2 groups based on Tönnis classifications. There was 1 T2 joint and 3 T3 joints. The T2 joint responded to treatment out to 2 years and 1 of 3 (33%) T3 joints responded to treatment at 6 months, none at 1 year and 2 years. All 3 of the T3 joints had joint replacement performed before the 1 year follow-up. 

### 3.4. 1st CM Joints

Both of the 1st CMC joints showed good results that remained through the 2-year follow-up. 

## 4. Discussion

This study is the first to show good clinical results using IAL in conjunction with PRP for arthrosis in humans. No complications or adverse events occurred. Overall 46% of joints had good clinical relief at six months post treatment and this good relief was sustained for 32% of joints out to 2 years. All of these improved patients were able to avoid surgery. SANE scores improved significantly in joints at 1 year and 2 years post treatment. For knee joints, which was the only subgroup large enough to test for clinical significance, there was significant improvement in SANE, KOOS-PS and WOMAC scores pre to post treatment. 

Our study is too small to draw many other conclusions regarding sub groups. However, in general, the results for joints with more severe pathology (i.e. BOB/1mm or T3) showed similar improvement to joints with greater joint space at six months, but these results deteriorated more at one and two years in the more severe pathology group than in the greater joint space group. Thus, this treatment regimen has been effective even in the most severely affected patients in the short term. Repeated treatment with PRP and laser may extend the benefits in this group for a longer period of time. 

The other sub group of note is the knees with patellofemoral joint arthritis. This group showed some of the highest positive results out to 2 years and no joint replacements performed. Although this group was small (4 joints), these results parallel our experiences with PRP in general and therefore, we believe that the results are not due to small group size but actually represent a group that responds extremely well to PRP treatment. 

For all of these patients, total joint replacement was the only reasonable alternative to this treatment regimen. Indeed, many of the failures of treatment went on to total joint replacement. It is important to note that of the 13 joint replacements that were performed after treatment, 11 of them occurred in joints with BOB/1 mm or T3 classified joints. Of those joints with greater joint space, only 2 (18%) went on to have joint replacement. 

This is the first study that has combined IAL and PRP for the treatment of osteoarthrosis. Studies in animals performed with surface LLLT and PRP have demonstrated healing in tendons and muscles and shown that the combination of laser and PRP can be more effective than either treatment alone [2,3]. The smaller size of the animals used in these studies may have contributed to the effectiveness of the treatment, since the laser light could pass more easily into the joints. We could find no other studies that have combined laser treatment with PRP in humans. PRP treatment alone for osteoarthrosis has been shown to be effective in multiple studies [15,16]. LLLT treatment results in humans have been more variable than in animals [4,5,6,7,8,9,10,11,12], possibly because of the greater joint size. By moving the laser intra-articularly, we minimized loss of light due to tissue obstruction and maximized the possible effectiveness. Combining IAL and PRP, this study produced results that showed the same type of synergy that was seen in animal studies. Our study used one 30-min laser treatment at the time of the PRP injection. This differs significantly from previous LLLT studies which use multiple treatments over several weeks. Huang’s systematic review [4] reports on 9 studies with 8 to 20 treatments over 2 to 6 weeks. The single intra-articular treatment used in this study appears to have worked as well or better than multiple LLLT treatments. It is unclear if this is because the light was applied intra-articularly, or because it was done in conjunction with PRP. 

While the mechanism of action of biophotomodulation is an area of continued research, it appears that the cytochrome-C oxidase molecule acts as a chromophore for red and near infrared light resulting in increased energy and nitric oxide production [17]. An increase in transforming growth factor beta may result. As for the synergy between IAL and PRP, we can only speculate that the additive effects of each are responsible for the benefit seen. Our goal in this study was to assess clinical efficacy. It is expected that in the future we will gain a greater knowledge of mechanisms of action.

The obvious weakness of our study design is the lack of a control group which would have had repeat PRP injection without IAL. Thus, we cannot rule out the possibility that some of our PRP-IAL patients would have improved with another PRP injection alone without IAL. However, in our prior extensive experience, now numbering over 3000 injections, we had generally not found subsequent PRP injection to be clinically effective if no improvement was seen by 1 month after the initial injection. Thirteen of the treated joints had had only 1 injection, while 17 had 2 or more initial injections without significant enduring clinical benefit. The outcomes between these two groups were not statistically different after the PRP IAL treatment. Therefore, we think it is unlikely that our current study patients would have benefited from repeat PRP alone without the addition of IAL. 

While we did not have a non-treatment control group, the six-month follow-up period for evaluation of efficacy is well established as a benchmark for true clinical benefit versus a placebo effect. This is the standard used for visco-supplementation injection. Patients with prolonged symptomatic arthrosis do not generally spontaneously significantly improve without additional treatment. Therefore, we believe that these clinical effects represent a true response to treatment. 

## 5. Conclusions

IAL in conjunction with PRP is very safe. It has significant efficacy in reducing arthrosis symptoms for up to two years in some patients who had failed prior PRP injection for the knee, hip, gleno-humeral and first carpo-metacarpal joints, especially those with moderate rather than bone on bone arthrosis.

## Figures and Tables

**Table 1:** Patient demographics.

Part	# of Joints	Mean Age	Male	Female
Knees	22	68.5	7	15
PF	4	65.8	1	3
Med	16	69.2	5	11
Lat	2	68.0	1	1
Hip	4	62.8	4	0
T2	1	60.0	1	0
T3	3	63.7	3	0
Shoulders	2	72.5	2	0
1st CM Jt	2	60.0	1	1
Total	30	67.4	14	16

**Table 2:** Summary of treated knee results.

Knees		All Knees	PF	Med	Lat	BOB & 1 mm	2 mm & up
**Total # of Joints**		22	4	16	2	12	10
**Pre-Treatment Scores**	**Mean SANE**	68.3	77.5	63.8	86	66.7	70.2
	**Mean KOOS-PS**	50.6	55.3	49.9	46.8	48.3	53.5
	**Mean Womac**	46.1	46.0	45.3	57.0	37.8	28.5
**6 Mo Totals**	**Total Joints**	22	4	16	2	12	10
	**# Good Outcome**	9	2	7	0	5	4
	**# Bad Outcome**	12	1	9	2	6	6
	**UK Out come**	1	1	0	0	1	0
	**% Good**	42.9%	66.7%	43.8%	0.0%	45.5%	40.0%
	**# of Jt Replac**	2	0	2	0	2	0
	**% Total Jt Repl**	9.5%	0.0%	12.5%	0.0%	18.2%	0.0%
	**Mean SANE**	47.0	34.0	47.7	78.0	29.3	40.0
	**Mean KOOS-PS**	64.0	71.7	63.2	48.8	20.5	58.7
	**Mean Womac**	20.8	-	20.8	-	3.3	4.4
**1 Year Totals**	**Total Joints**	22	4	16	2	12	10
	**# Good Outcome**	6	2	4	0	2	4
	**# Bad Outcome**	15	1	12	2	9	6
	**UK Out come**	1	1	0	0	1	0
	**% Good**	28.6%	66.7%	25.0%	0.0%	18.2%	40.0%
	**# of Jt Replac**	6	0	6	0	6	0
	**% Total Jt Repl**	28.6%	0.0%	37.5%	0.0%	54.5%	0.0%
	**Mean SANE**	40.0	7.5	47.2	-	15.4	25.5
	**Mean KOOS-PS**	65.6	89.6	61.8	56.0	20.5	54.1
	**Mean Womac**	21.5	9.0	24.0	-	4.0	8.1
**2 Year Totals**	**Total Joints**	22	4	16	2	12	10
	**# Good Outcome**	6	2	4	0	2	4
	**# Bad Outcome**	15	1	12	2	9	6
	**UK Out come**	1	1	0	0	1	0
	**% Good**	28.6%	66.7%	25.0%	0.0%	18.2%	40.0%
	**# of Jt Replac**	9	0	8	1	7	2
	**% Total Jt Repl**	42.9%	0.0%	50.0%	50.0%	63.6%	20.0%
	**Mean SANE**	35.0	17.5	40.8	-	10.8	15.0
	**Mean KOOS-PS**	66.8	100.0	61.3	-	8.7	36.3
	**Mean Womac**	24.9	1.0	28.8	-	6.8	9.3

## Supplementary Materials

The following are available online at https://www.mdpi.com/2305-6320/6/3/75/s1, File S1: Patient Raw Data.
